# Screening for *in planta* protein-protein interactions combining bimolecular fluorescence complementation with flow cytometry

**DOI:** 10.1186/1746-4811-8-25

**Published:** 2012-07-12

**Authors:** Kenneth Wayne Berendzen, Maik Böhmer, Niklas Wallmeroth, Sébastien Peter, Marko Vesić, Ying Zhou, Franziska Katharina Elisabeth Tiesler, Frank Schleifenbaum, Klaus Harter

**Affiliations:** 1 Universität Tübingen, ZMBP, Plant Physiology, Auf der Morgenstelle 1, D-72076, Tübingen, Germany; 2 University of California, San Diego, Division of Biological Sciences, Cell and Developmental Biology Section & Ctr for Mol. Genetics 0116, 9500 Gilman Drive #0116, La Jolla, CA, 92093-0116, USA; 3 Universität Tübingen, ZMBP, Biophysical Chemistry, Auf der Morgenstelle 18, D-72076, Tübingen, Germany

**Keywords:** FACS, BiFC, *In planta*, *In vivo*, Protein-protein interaction screen, CPK3

## Abstract

Understanding protein and gene function requires identifying interaction partners using biochemical, molecular or genetic tools. In plants, searching for novel protein-protein interactions is limited to protein purification assays, heterologous *in vivo* systems such as the yeast-two-hybrid or mutant screens. Ideally one would be able to search for novel protein partners in living plant cells. We demonstrate that it is possible to screen for novel protein-protein interactions from a random library in protoplasted *Arabidopsis* plant cells and recover some of the interacting partners. Our screen is based on capturing the bi-molecular complementation of mYFP between an YN-bait fusion partner and a completely random prey YC-cDNA library with FACS. The candidate interactions were confirmed using *in planta* BiFC assays and *in planta* FRET-FLIM assays. From this work, we show that the well characterized protein Calcium Dependent Protein Kinase 3 (CPK3) interacts with APX3, HMGB5, ORP2A and a ricin B-related lectin domain containing protein At2g39050. This is one of the first **random***in planta* screens to be successfully employed.

## Background

Identifying interaction partners for proteins and expanding the list of known gene products that interact with a particular protein are crucial to studying protein function. Several methods exist for searching for novel protein interaction partners in an unbiased way, for example yeast two-hybrid [1], split-ubiquitin [2], and complex yeast screening assays [3]. Yet, while these methods are very useful, few attempts have been made for establishing library-scale screens *in planta*. However, an *in planta* screening method is potentially more reliable in regards of minimizing unspecific behaviors observed in heterologous systems, should allow for proper protein modifications, and presumably lead to discovering more functionally relevant interaction partners. A non-random library *in planta* screen has been developed using the split-luciferase system and a high-throughput 96-well protoplast transformation method [4]. It relies on screening defined plasmid pools, making it possible to determine many interactions in a small amount of space.

Other protein complementation assays exist that would lend themselves also to the establishment of high-throughput assays besides split-luciferase, for example dihydrofolate reductase (DHFR), split-ubiquitin and bimolecular fluorescence complementation (BiFC) [5,6]. BiFC generates fluorescence derived from the association of fragments of a fluorescent protein that are fused to interacting proteins once brought within proximity of one another. BiFC has been heralded as a very robust and reliable method for the detection of novel protein interactions *in vivo*, some of which can occur *via* intermediate complex-associated proteins and not direct binding [6,7]. An attempt at using BiFC in a ‘high-throughout’ *in planta* screen was used for testing 58 core cell cycle proteins [8,9]. This screen however was conducted in tobacco epidermal cells and is not suited for screening hundreds or thousands of interactions. Coupling of BiFC with flow cytometry has been shown to be a very sensitive method for screening applications, for detecting weak interactions between SH3 domains and partners in bacteria [10], and for plant cells [11-13].

Here, we present a method for the identification of unknown protein-protein interactions that occur *in planta*. This method is based on the detection of capture of YFP-BiFC emission by FACS between a bait fusion protein and a random fusion library. We have used protoplasts from *Arabidopsis* dark-grown cell culture, but the method should be applicable for protoplasts derived from any tissue. Establishing the method required testing of different YFP-fragment fusions for both bait and library, as well as determining flow cytometric detection limits. We illustrate our observations and present an example screen along with interaction confirmation using independent *in vivo* BiFC measurements, and *in vivo* FRET-FLIM measurements. We conclude with a thorough discussion of the results, including the advantages, disadvantages and possible screening improvements.

## Results

### Screen design

#### Brief summary

**Figure 1:**
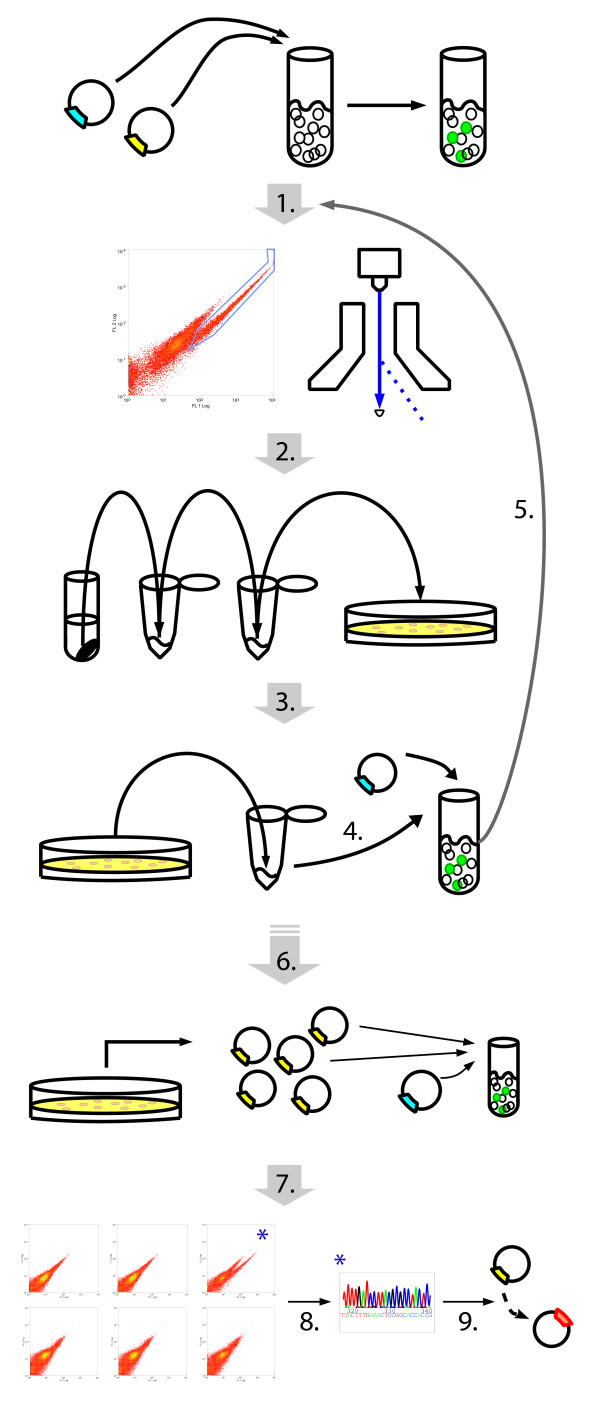
**Design for performing*in vivo*BiFC screens by DNA transfection in protoplasts.** The screen design is explained in detail in the text. In brief, DNA from a random library and specific bait are co-transfected into protoplasts. Protoplasts providing positive BiFC signals are identified and sorted by FACS. The DNA is harvested and the bacterial plasmids are recovered. The process is repeated with the new prey pool until a suitable number of putative bacterial colonies are obtained for further processing.

The novel *in planta* protein-protein library screen using BiFC technology is depicted in Figure1. The screen is based on recovering plasmid DNA from a random, plasmid encoded cDNA library that has been transfected along with a bait plasmid into living plant protoplasts. Protein interactions are observed in whole cells by detecting complemented YFP using a flow cytometer and are collected by Fluorescence Assisted Cell Sorting (FACS). Transfected plasmid DNA that is present in the collected protoplasts is isolated and transformed into bacteria. Plasmids from these bacteria are re-isolated, pooled and transfected again with the bait-plasmid into plant protoplasts; positives are identified and collected as just described. From there, plasmids from individual bacterial colonies are tested against the bait for BIFC in plant protoplasts. The plasmid DNA from those transfection events with positive BIFC signals are then sequenced to identify the cloned cDNA whose encoded proteins represent the set of putative interactors with the bait protein.

#### The screen

The optimized screen is shown in Figure1 and described in the following text in detail. As we worked with *Arabidopsis thaliana*, we recombined an *Arabidopsis* third-flower stage seedling cDNA library into the pE-SPYCE plasmid (see Methods for specific details) which carries an ampicillin bacteria resistance marker. The coding-sequence of a bait of interest is cloned into a plasmid to create a BAIT-YN fusion protein that has a bacterial marker other than ampicillin. In our case, we used a bait plasmid that carried a spectinomycin bacterial marker and an YN173 BiFC fragment. The location of the tag, C- or N-terminus is dependent on the protein of interest, but for this work, the bait fusion was C-terminal. Once the cloning is completed, the plasmids need to be highly purified and concentrated; for example, by cesium chloride purification or an equivalent method.

For this screen, 5 μg of the purified bait plasmid was co-transfected along with 15 μg purified pPE-SPYCE-cDNA library plasmid DNA per transfection into protoplasts (Figure1.1). We generated protoplasts from *Arabidopsis* cell-culture as described in [12]. The transfected protoplasts were incubated for 24 to 36 h at 26°C in the dark. After the incubation period, the protoplasts with a positive BiFC signal were detected and sorted by FACS. The maximum number of BiFC positive cells was obtained by plotting the primary YFP fluorescence channel against the secondary YFP fluorescence channel and choosing those cells that had significant shifts in the YFP channel over the autofluorescence. Exact FACS parameters can slightly vary between machine set-ups, but any protocol that approximates our protocol should work (see Methods for FACS details).

The protoplasts were sorted directly into a 2 ml eppendorf tube containing 300 μl Edwards DNA extraction buffer [14] at 20°C (Figure1.2). The collected protoplasts were thoroughly mixed, iso-propanol was added at a 1:1 ratio and mixed, followed by incubation at −20°C for 30’ to precipitate DNA, centrifuged at 13000 rpm at 4°C for 45’ on a bench-top micro-centrifuge, washed 500 μl with cold 75% ethanol, centrifuged again at 4°C for 15’ and the resultant precipitate was air-dried for 15’ on the bench at room-temperature to be finally resuspended in 20 μl Millipore purified water.

The purified DNA extracted from the protoplasts was transformed into highly chemically competent bacterial cells and selected on ampicillin at 28°C to select for library plasmids (Figure1.3). Specifically, chemically competent NEB 10-β (New England Biolabs) cells were used as they have been optimized for high transformation efficiency with large plasmids and the library plasmid without an insert is ~8 kb. Fifty micro-liters of NEB 10-β cells were thawed on ice and added to 10 μl of the DNA precipitate, followed by an incubation at 4°C for 30’ then a 30” heat-shock at 42°C, 2 min incubation on ice, and a longer incubation for 2 hrs at 37°C in 800 μl SOC medium with shaking. The transformation was plated out on two large (145 x 20 mm) Petri dishes containing LB-agar with 100 μg /ml ampicillin and placed at 28°C to select for colonies with the pE-SPYCE-cDNA library vector.

To recover plasmid from colonies on a plate, the bacteria were mixed and removed in 10 ml LB directly to the plate; the LB contained selection antibiotic. Plasmid DNA was isolated using a DNA Maxi-prep kit and transfected into protoplasts along with the bait-plasmid (Figure1.4) as described above. The positive BiFC cells were sorted by FACS (Figure1.5) and processed just as described to isolate bacteria transformed with the library plasmid.

Plasmid DNA from single colonies was tested individually after this second round of protoplast transfection / FACS / bacteria transformation. The plasmid DNA from each colony was purified using commercial midi-DNA preparation columns. This plasmid DNA from each colony was transfected individually along with the bait encoding plasmid (Figure1.6) and screened for BIFC by flow cytometry (Figure1.7). BiFC expressing cells identified from this analysis were those carrying plasmids encoding the putative bait interactors. The plasmid DNA from those positive colonies was sent for sequencing (Figure1.8) and cDNAs were re-cloned into virgin plasmids (Figure1.9) for confirming the interaction and continued analysis.

The screen takes about 3 to 4 weeks to positively identify individual colonies. The difficult and more time consuming part is the obligatory re-cloning of the cDNAs. Repeated attempts at rescuing the cDNAs from the isolated pE-SPYCE vectors by BP recombination reactions failed. As there were typically multiple plasmids inside each colony, visible in the DNA sequence trace files as minor peaks (Additional File 1), one might presume that this interfered with the recombination reaction, but this was not confirmed. Nevertheless, a dominate sequence could be identified for most of the positive signals, and this sequence corresponded to a clear, singular cDNA sequence as determined by BLAST analysis against the *Arabidopsis* cDNA banks. Thus, this dominate sequence was presumed to encode for the interacting partner. According to our data this was the correct assumption (see case study screen below). The cDNAs encoding for the putatively interacting partners could be amplified by PCR either from the recovered plasmid DNA, the original pPE-SPYCE-cDNA library or from freshly won cDNA from *Arabidopsis* leaf material. The screen ends in the confirmation of the BiFC interactions using alternative methods.

### BiFC sensitivity

#### Cytometric sensitivity limit

**Figure 2:**
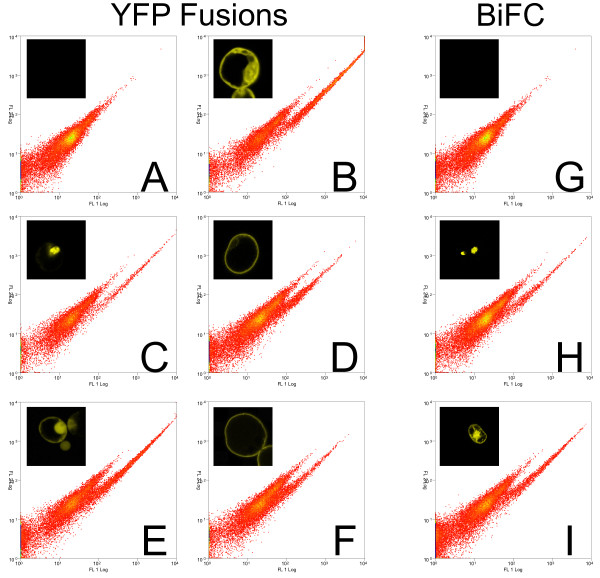
**Detection sensitivity of YFP fusion proteins.** We tested our cytometer for its detection sensitivity of different protein fusions. One can see from comparison with the mock negative control, that YFP expressing protoplasts can be clearly identified as a cloud of cells shifted to the right in the FL1 channel. **A.** Mock transfected (water only), **B.***CPK3-mYFP***C.***ARR2-mYFP***D.***FLS2-mYFP***E.***free mYFP***F.***SLAC1-mYFP***G.** Mock transfected (water only), **H.***bZIP63-YN + bZIP63-YC*[6], **I.***T14-3c-YN + T14-3c-YC*[6].

BiFC fluorescence signals are known to be less bright compared to non-truncated mYFP [15]. Therefore, full-length YFP and YFP derived BiFC signals were tested to ascertain the detection limit of our FACS system (Figure2). We tested previously described proteins as full length protein–mYFP fusions. Besides testing well-known homomeric proteins such as bZIP63 and T14-3c that are known to make specific and strong BiFC [6], we also tested two well-known plasmalemma localized proteins: SLAC1(SLOW ANION CHANNEL-ASSOCIATED 1) [16] and FLS2 (FLAGELLIN-SENSITIVE 2) [17], as well as an additional nuclear localized protein, ARR2 (ARABIDOPSIS RESPONSE REGULATOR 2) [18].

Free mYFP and the different mYFP fusion proteins, including those restricted to the plasma membrane, were clearly detectable by FACS for all of the tested fusion proteins. BiFC derived signals of bZIP63 and T14-3c could be detected by FACS and these are localized to the nucleus and cytoplasm/nucleus, respectively (Figure2).These results indicated that all of the full-length mYFP-fusion proteins could be detected by flow cytometry and this observation should be applicable to other proteins with the same intercellular distributions. BiFC expression is estimated to be 10x weaker than the full-length mYFP but the homodimers of bZIP63 and T14-3c were well detected. As a note, our results are limited to our FACS set-up, but should be good starting points for other laboratories using similar technology.

#### Screening specificity and background signals

**Figure 3:**
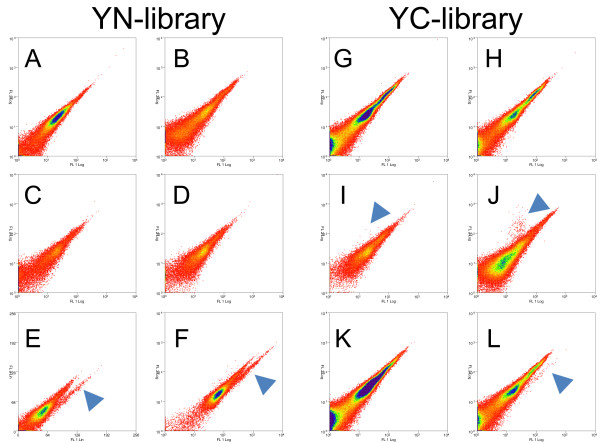
**Screening controls for the YN-cDNA and YC-cDNA libraries.** X-axis is YFP fluorescence and Y-axis is autofluorescense; arrows show and indicate positive mYFP-BiFC signals. **A.** Mock transfected cells. **B.** Cells transfected with *YN-Library* alone. **C.** Cells transfected with *free YN* fragment alone. **D.** Cells co-transfected with *YN-Library* and *free YC* fragment. **E.** Cells co-transfected with *bZIP63-YC* and *YN-Library*; a clear YFP-BiFC signal was detected. **F.** Cells co-transfected with *mYFP-YC* and *YN-Library*; a clear YFP-BiFC signal was detected. **G.** Mock transfected cells. **H.** Cells transfected with the *YC-Library* alone. **I.** Cells co-transfected with *mRFP-YN* and *YC-Library;* only mRFP signal was detectable. **J.** Cells co-transfected with *mCherry-YN173* and *YC-Library*; only mCherry signal was detectable. **K.** Cells co-transfected with *bZIP63-YN* and *YC-Library*. **L.** Cells co-transfected with *CPK3-YN173* and *YC-Library*; a clear YFP-BiFC signal was detected.

BiFC interactions are purported to be irreversible [7] and require many tests to control for spontaneous association of the YFP fragments [15]. Techniques designed for reducing non-specific background in confocal set-ups [15] were not suitable for designing this screen to capture rare, weak signals. Therefore we used both the *YN154* and *YC155* fragments to generate *Arabidopsis* cDNA libraries. These libraries were then tested to identify which library that would generate BiFC signals that could be attributed to specific protein-protein interactions (Figure3). The first library that we generated and tested was with the YN fragment. The *YN154* fragment in fusion with cDNAs or alone, or in combination with free YC fragments did not show any BiFC signals in transfected protoplasts (Figure3B, C, D). While seemingly encouraging, the free YN fragment would however spontaneously associate when the YC fragment was fused to any protein as exemplified by spontaneous association of free YN with bZIP63-YC or a non-functional control, mRFP-YC (Figure3E, F). This is best explained by the fact that while bacterially purified YC fragments are mostly insoluble [19,20] and this is likely the case with free YC fragments *in vivo* as well, the YN fragment is stable enough to cause some non-specific complementation. Although we thought that perhaps the non-specific background would be drowned out by specific bait interactions detectable in flow cytometry as strong fluorescence signals, a preemptive screen using the *YN154-cDNA-library* taking only strong BiFC signals detected by FACS from a bZIP63-YC / *YN154-cDNA-library* co-transfection, resulted in the identification of BiFC signals that were derived from empty or out-of-frame *YN154-Library* plasmids (not shown). This indicated that the screen could not be conducted in this orientation.

In light of the knowledge gained from the YN-library, the YC-library was expected to perform better, since any empty or out-of-frame plasmids should not give any BiFC signal. Indeed this was the case, as only YC fusions, and not just YC fragments, resulted in positive BiFC signals using the large-scale transformation method (Figure3G-L). What was surprising, however, was that we were not able to obtain a BiFC signal with bZIP63-YN / YC-Library co-transfections. Therefore, a longer YN-fragment including the 8^th^-beta strand (YN173) known to have better complementation efficiency [21] and is therefore brighter, was generated in order to overcome this deficiency. We did not take brighter YFPs derivates such as VENUS or mCitrine due to their higher rates of spontaneous BiFC association [15,20] as they would have led to unspecificity during the screen. An additional control fusion construct *YN173-mCherry* was made and also tested against the *YC-library*. Neither the *YN154-mRFP* nor the *mCherry-YN173* co-transfection with *free YC* or the *YC-Library* lead to any non-specific BiFC (Figure3I, J). Thereafter, we decided in addition to using the brighter *YN173* fragment that it would be helpful to take a protein whose expression domain is more widespread than bZIP63 which is only found in the nucleus. Therefore, we choose a protein, Calcium Dependent Protein Kinase 3 (CPK3; AT4G23650) that is fairly well characterized and known to be present in both the cytoplasm and the nucleus and interact with proteins in both compartments [22,23]. While the CPK3-YN173 fusion protein showed no YFP fluorescence by itself in transfected protoplasts, clear BiFC derived signals were observed when *CPK3-YN173* was co-transfected along with the *YC-Library*. Additionally, the lack of any detectable interactions of the YC-library with YN154-mRFP and YN173-mCherry suggested that the interactions observed by CPK3-YN173 were due to specific interaction with YC protein fusions from the library. These results indicate that the screening conditions had been met: the detection of specific interactions in a rare-event analysis. We therefore followed CKP3-YN173 through a complete screen as it fulfilled all prerequisites required for a successful BiFC screen (Figure3).

### Screening the YC-library: screening with CPK3, a case study

#### Screening results

**Figure 4:**
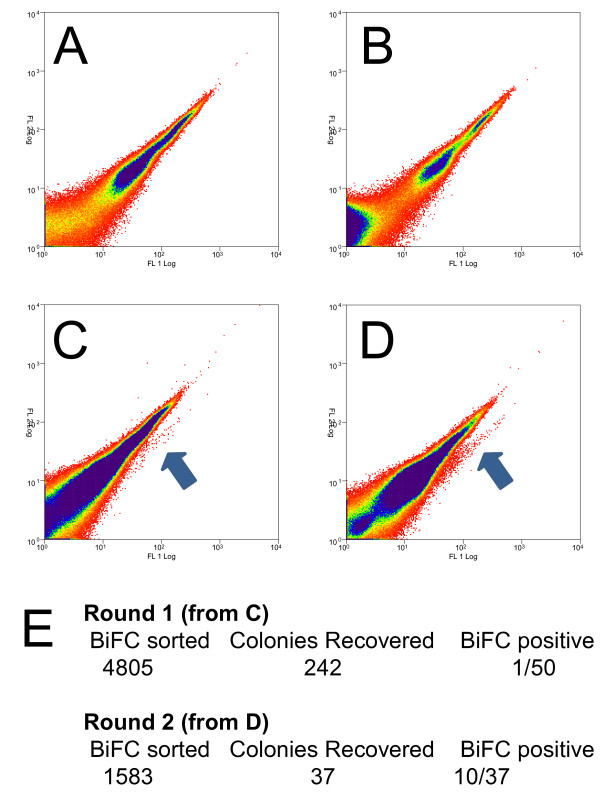
**Cytometric and plasmid recovery results from the CPK3 screen.** Panels **A**- **D** show typical results of observing 2 x 10^5^ to 4 x 10^5^ events; more than 2.5 x 10^6^ events were analyzed per FACS session. **A.** Mock transfected (water only), **B.***CPK3-YN173* transfected alone (no YFP signal is produced), **C.** First Round, initial co-transfection of *CPK3-YN173* and *YC-cDNA* library, **D.** Second Round, co-transfection of the fished library plasmids of the first round, pooled challenged with bait *CPK3-YN173*, **E.** Number of positive BIFC cells sorted, the number of recovered bacterial colonies and the number of colonies producing positive BIFC in one-on-one challenges with the bait (actual plots are showed in Figure5).

The screen with bait CPK3-YN173 was conducted according to Figure1. The CPK3-YN173 fusion was negative for self-complementation (figure3K, 4A). Furthermore, BiFC signals were detected in the protoplast population, when *CPK3-YN173* was co-transformed with the *YC*-cDNA library. 2.5 x 10^6^ transfected protoplasts were screened for BiFC signals resulting in 4805 sorted events in the first round (figure3L, 4B). Two-hundred and forty-two (242) bacterial colonies were obtained from the DNA preparation of these 4805 sorted protoplasts, a ‘recovery-rate’ of 5%. Of those, only 1 out of 50 tested by flow cytometry in pair-wise challenges with the bait showed a positive BiFC signal (i.e. only 2%). Therefore, the plasmid DNA was purified from all 242 colonies and challenged with the bait plasmid in protoplasts to enrich for plasmids that carry protein fusions that specially interact with the CPK3-YN173 bait. The second round of 2.5 x 10^6^ transfected protoplasts resulted in 1588 positive BiFC events from which 37 colonies were obtained, a ‘recovery-rate’ of 2%. However, this time significantly more YC fusion constructs from singular colonies lead to positive BiFC events with the bait construct were recovered: 10 out of the 37 had positive BiFC signals, meaning that 27% had encoded fusion proteins putatively interacting with the bait (figure4B, 5A). Thus, a total of eleven colonies had been found containing plasmids encoding for interaction partners with the CPK3-YN173 bait fusion protein whose in-frame fusions were confirmed by DNA sequencing. From these 11 plasmids, only 8 delivered readable DNA sequence trace files, as the other three had multiple plasmids inside as judged by strongly overlapping peak trace signals. From those 8, a clear sequence corresponding to a specific *Arabidopsis* ORF was identified by BLAST although there were minor sub-traces in some of the sequence traces (Additional File 1). This indicated that although a major plasmid species had been discovered present in each bacterial colony, the bacteria still picked up other plasmids as well. The majority of the ORF-matching sequences that were obtained included or started near the start-codon. The prey inserts were recalcitrant to re-cloning into expression vectors via Gateway™ recombination which would have allowed us to analyze a single plasmid species; thus, we were not able to determine the complete ORF coverage for all of the clones. Nevertheless, the sequences were sufficient for assigning gene identity. Minor plasmid species present in the same bacterial colony were assumed to be off-targets. We therefore decided to clone the full-length ORF of each gene coding for each putative interaction partner using the corresponding cDNAs obtained from *Arabidopsis* tissue. The putative interacting proteins of CPK3-Y173 are listed in Table1.

**Figure 5:**
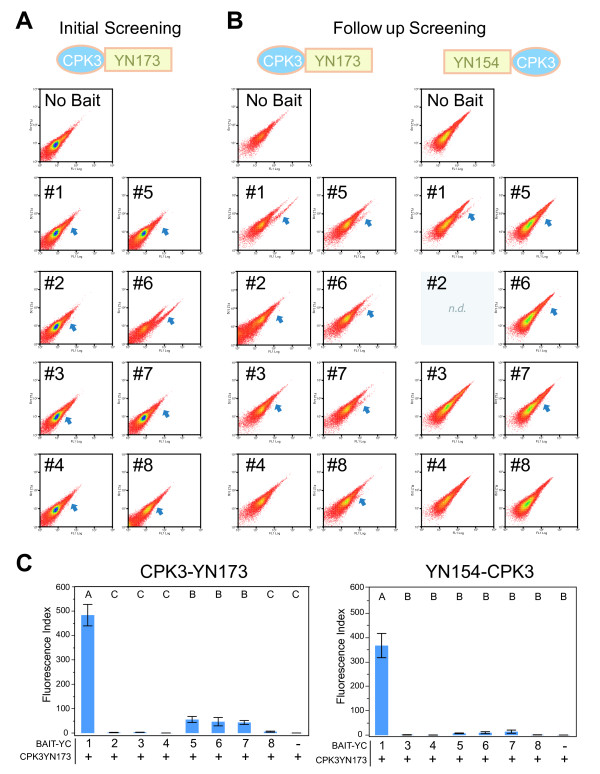
**Cytometric plots of testing for BIFC between bait CPK3-YN173 and fished YC fusion proteins.A.** Cytometric plots are shown from the second round screening that lead to 8 positive colonies (3 were eliminated due to technical difficulties, see text). **B.** Subsequent testing of the 8 candidate proteins after re-cloning of the candidate cDNAs and tested against CPK3-YN173 or YN154-CPK3. **C.** The YFP BiFC index was calculated as previously published [12]; it is the mean times fluorescence intensity. These experiments were repeated in their entirety 3 times with 3 replicates per experiment with similar results. Colony #2 was delayed in cloning and therefore showed with its respective negative free-SPYCE control. n.d. = not done.

**Table 1:** Table of putatively CPK3 interacting proteins

**Colony Number**	**AGI Identifier**	**Common Name**	**Description**
1	AT4G35000	APX3	Encodes a microsomal ascorbate peroxidase; scavenges hydrogen peroxide
2	AT2G29670	AT2G29670	tetra-trico peptide repeat (TPR) containing protein
3	AT5G08680	AT5G08680	Encodes one of three mitochondrial ATP synthase beta-subunits
4	AT3G14420	GLO1	miscrosomal glycolate oxidase, involved in the production of hydrogen peroxide
5	AT4G22540	ORP2A	oxysterol binding protein (OSBP); sterol trafficking, affecting membrane fluidity and permeability and influencing secretory events
6	AT2G39050	AT2G39050	ricin B-related lectin domain containing protein; traffics from the ER, via the Golgi complex, to the vacuole
7	AT4G35570	HMGB5	chromatin-associated protein that binds to the minor groove of short stretches of A/T-rich B-form DNA
8	AT1G67980	CCoAMT	Encodes S-adenosyl-L-methionine: transcaffeoyl Coenzyme A 3-O-methyltransferase

**Figure 6:**
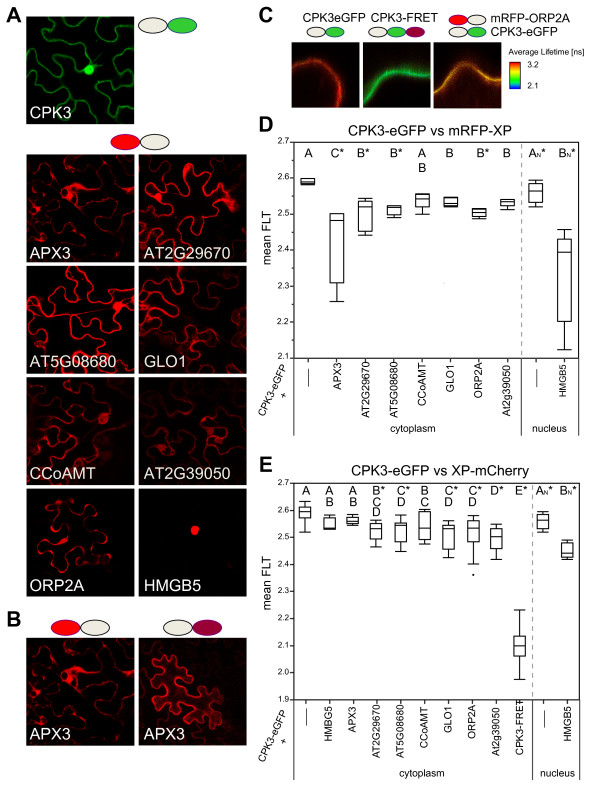
**Results of FRET-FLIM measurements of CPK3 with candidate interaction proteins.A.** Example expression domains of CPK3-eGFP and mRFP-XP fusion proteins as seen in tobacco epidermal cells. **B.** Comparison of the localization pattern of APX3 with either an N-terminal mRFP fusion (left) or a C-terminal mCherry fusion (right). The unhampered fusion is the N-terminal mRFP fusion which shows an uneven expression domain unlike the C-terminal mCherry fusion. **C.** Example of false-colored FRET-FLIM imaging sectors. Stronger FRET-FLIM results in a reduction in the average lifetime. The negative control in CPK3-eGFP alone; the positive control is a CPK3-eGFP-mCherry (called CPK3-FRET) fusion; and real sample of CPK3-eGFP co-expressed with mRFP-ORP2A. **D.** The averages of the average lifetime (from the measurement sector) are shown as box plots for the CPK3-eGFP in the presence of mRFP-XP fusions. Letters are significance classes based on Students t-test; any sample not connected by a letter is significantly different from the CPK3-eGFP control. Significance difference to the control using Dunnett’s Method is indicated by a *. **E.** The averages of the average lifetime (from the measurement sector) are shown as box plots for the CPK3-eGFP in the presence of XP-mCherry fusions. Letters are significance classes based on Students t-test; any sample not connected by a letter is significantly different from the CPK3-eGFP control. Significance difference to the control using Dunnett’s Method is indicated by a *. Cytoplasm and Nucleus indicate in which compartment the measurements were made.

The colony number as in Figure5A is given for consistency along with the gene AGI identifier and common name of the plasmid predominately present in the plasmid prep. The dominate cDNA sequence was re-cloned from fresh Arabidopsis cDNA for confirmatory BiFC (data in Figure5B, C) and FRET-FLIM experiments (data in Figure6).

YC clone 1 (AT4G35000) contained the *ascorbate peroxidase 3* (APX3) cDNA. APX3 has been shown to localize to the peroxisomes and to be involved in H_2_O_2_ detoxification [24]. YC clone 2 (AT2G29670) carried a cDNA encoding a tetratrico peptide repeat (TPR) containing protein. TPRs mediate protein-protein interactions in a wide variety of cellular processes [25]. The cDNA insert of clone 3 (AT5G08680) encoded a mitochondrial ATP synthase (EC 3.6.3.14) beta-subunit that has been previously purified along with mitochondria [26]. The insert of clone 4 (AT3G14420) encoded a putative glycolate oxidase (GLO1), that has also been co-purified with peroxisomes [27] and is involved in H_2_O_2_ production. Clone 5 (AT4G22540) encoded o*xysterol binding protein-related protein 2A* (ORP2A). Oxysterol binding proteins (OBPs) are thought to control sterol traffic between membranes [28]. Clone 6 (AT2G39050) carried a cDNA encoding a ricin B-related lectin domain that is commonly associated with membranes [29]. Clone 7 (AT4G35570) encoded the *high mobility group B protein 5* (HMGB5). HMGB5 is a chromatin associated protein involved in controlling DNA architecture influencing transcription [30]. Finally, clone 8 (AT1G67980) encoded an enzyme with putative caffeoyl-CoA O-methyltransferase activity (CCoAMT, EC 2.1.1.104). It is most likely involved in the phenylpropanoid pathway. Quite remarkably, many of these fished proteins have domains that are membrane associated.

#### BiFC validation of putative CPK3 interaction partners

To validate and quantify the BiFC interactions, the identified prey cDNAs were cloned to virgin YC-fusion plasmids and individually tested against YN-CPK3 fusions under the conditions as those used for the screening. After re-cloning of all of the cDNAs into the different vectors, their potential for generating positive YFP derived BiFC was tested pair-wise against the bait versions in small-scale transfections (Figure5B).

The screen was made with the YN-fragment fused to the C-terminus of CPK3. It is known CPK3 can be myristoylated at the N-terminus and associates with membranes [23,31,32]. However, CPK3 is also known to be present in or around the nucleus [22,23]. CPK3-mYFP localized in the cytoplasm and in and around the nucleus in transiently transformed *Arabidopsis* protoplasts and *Nicotiana benthamiana* epidermal leaf cells under our conditions (Figure2; Figure6). The recorded fluorescence pattern does not exclude the specific association of CPK3 with membranes or other cell compartments nor is it different from previous publications. Nevertheless, it cannot be excluded that the cloning linker interfered with CPK3´s myristoylation. We, therefore, also tested an YN154-CPK3 fusion that definitely masks the CPK3 myristoylation site to appraise its effect on the interactions with the fished genes and compared it to the CPK3-YN173 version.

Three biological replicates were done with three technical transfection replicates per experiment for quantifying BiFC fluorescence by analytical flow cytometry. The same trend was observed in all three experiments. Detailed statistical results for Student’s t-test and Dunnet’s test are given in Additional file 2. The cDNAs of clone 2 (AT2G29670), 3 (AT5G08680), 4 (GLO1) and 8 (CCoAMT) produced no BiFC with CPK3. In contrast, clone 6 (AT2G39050), 5 (ORP2A), and 7 (HMGB5) and showed some weak (and occasionally significant) BiFC with CPK3. On the other hand, clone 1 (APX3) interacted very strongly and irrefutably with CPK3 (Figure5C). These interactions, as measured by quantified BiFC, were observed for the CPK3-YN173 as well as for the YN154-CPK3 construct, albeit that the YN154-CPK3 signals were radically weaker overall compared to CPK3-YN173. For those proteins that showed no BiFC, no detectable protein was observed by western blotting, and it is presumed these proteins were not expressed in the protoplasts derived from the *Arabidopsis* dark-grown cell culture for unknowable reasons although the interaction conditions were maintained the same as the screening conditions.

#### Interaction validation with FRET-FLIM

To substantiate the BiFC protein-protein interactions *in planta,* fluorescence resonance energy transfer – fluorescence lifetime (FRET-FLIM) measurements were carried out. This was also necessary as BiFC complexes are supposedly irreversible [7] and could form from non-specific interactions [15]. FRET only occurs over a limited range of 100 Å, and is theoretically only possible when there are actual protein-protein associations [33]. Thus FRET-FLIM allows one to determine if associations are real, *in vivo* protein-protein interactions and reveal how dynamic or transient the associations are. To study FRET-FLIM, we switched from the *Arabidopsis* protoplast system to tobacco leaves using a transient *Agrobacterium* transformation method.

We used the pABind; [34] vector set that involves cloning cDNAs into a C-terminal donor (eGFP) or a C-terminal mCherry. In addition, we fused the fished proteins to an N-terminal mRFP [35] to mirror the screening orientation. Simply put, GFP is excited and if it is near mRFP or mCherry, FRET from the GFP to the mCherry molecule results in a reduction of the GFP’s fluorescence lifetime [33]. The lifetime of eGFP is then measured within a small window from which one can calculate the average fluorescence lifetime (Figure6C). The experiment was repeated for at least five independent cells per sample. The expression of the fusion protein was induced by estradiol application (Figure6A, B).

We had previously shown that free proteins did not exhibit any non-specific FRET-FLIM of donor to acceptor [35]. We first confirmed that the CPK3-eGFP localized to the cytoplasm and the nucleus (Figure6A) as all of the proteins interacting in BiFC were localized to these compartments (Figure6A). FRET-FLIM measurements were conducted in a cytoplasmic space, except for HMGB5 that was exclusively localized in the nucleus (see Additional file 3 for comparison of mCherry and mRFP fusions).

We first tested for FRET-FLIM using the same fusion protein orientations as those used in the screen. Detailed statistical results for Student’s t-test and Dunnet’s test are given in Additional file 2. According to the FRET-FLIM data and using Student’s t-test (α = 0.05), all samples except for CCoAMT caused a significant reduction in the fluorescence lifetime of GFP. A more stringent significance test, Dunnett's Method showed that a fraction of those were significantly different. If we restrict ourselves to Dunnett’s Method, then APX3, AT2G29670, AT5G08680, ORP2A, and HMGB5 all showed FRET-FLIM reductions with CPK3. APX3 and HMGB5 interacted strongest with CPK3 as they showed the greatest reduction in the fluorescence lifetime of the GFP donor.

We also tested the CPK3-eGFP for FRET-FLIM with the putative interactors C-terminally fused to mCherry (Figure6E). AT2G29670, AT5G08680, GLO1, ORP2A, AT2G39050, and HMGB5 showed significant reductions in the GFP fluorescence lifetime, indicating that they also interacted with CPK3-GFP. In contrast to the N-terminal mRFP fusion with does interact with CPK3-eGFP, the C-terminal mCherry fusion of APX3 (APX3-mCherry) did not. This could be due to a block of APX3´s C-terminal peroxisome targeting motif [24] by mCherry. Interestingly, compared to mRFP-APX3, APX3-mCherry was more uniformly distributed in the cytoplasm (also see Additional file 3). Thus, APX3-mCherry is presumably not able to associate with microsomal compartments any longer (Figure6B). Based on these observations, APX3 requires its C-terminal targeting signal for interaction with CPK3.

We performed additional negative controls by measuring the fluorescence lifetime of CPK3-eGFP in the cytoplasm when HMGB5 was present in the nucleus. As shown in Figure6E no significant reduction was observed (Figure6E). Summed together, APX3, ORP2A and HMGB5 all interacted via BiFC and FRET-FLIM with CPK3 under the same screening orientation with RTB-like AT2G39050 also being a suitable candidate.

#### Interaction validation with yeast-2-hybrid

To further support the idea that interacting proteins were found, we also tested the putative CPK3 interactions with the yeast-two-hybrid assay. We were not able to validate any of the interactions in the yeast-two-hybrid assay (Additional file 4). We repeated the transformation four times and at different temperatures (28°C and 16°C) but we could not obtain any interaction. Furthermore, the strongest candidate, APX3 was lethal to the yeast cells as very few colonies were obtained at each transformation and for those that were transformed, they did not express the protein (Additional file 4). Other than APX3 and AT5G08680, all of the putative interacting proteins were expressed in the yeast system. Although perhaps alternating the tags in the yeast system or manipulating the screening environment might be helpful to rule out any other over-expression effects of the fished proteins, we conclude that the proteins found to interact with CPK3 in this screen cannot be routinely detected in a standard yeast-two-hybrid assay.

## Discussion

### Challenges of the *in vivo *BiFC screen

We have showed that it is possible to find proteins from a random library that interact with a bait protein *in vivo* and *in planta.* This was done by observing protein-protein interactions measured by BiFC of mYFP. Although this screen is one of the few purely random *, in planta* screens ever established, its efficacy is not perfect. We had to come up with a method that would allow the identification of a single population of interacting proteins from a single protoplast. We used several techniques that were impossible to avoid but are difficult to control: transfection of protoplasts, recovery of plasmids from protoplasts and transformation of bacteria with plasmid. The first condition is obvious: in order to make a screen in living plant tissue, it is necessary to have a method that has the capacity of performing many interactions simultaneously and we choose protoplast transfection. However, there is no way to exclusively introduce a single plasmid into a protoplast, the nature of the method means that each protoplast takes up multiple plasmids. Thus methods that would rely on PCR (single cell PCR) for single cells or pooling of the cells (454 sequencing) would most likely not eliminate false-positives. An attempt to dilute the mixed library DNA to some statically desirable transfection rate of 1:1 of bait to prey would be dependent on the transfection rate, which varies from batch to batch, and most likely produce undetectable protein amounts. An alternative to our random library approach would be to use defined grids of prey plasmids that could be transfected into the protoplasts as binary pools; such an approach was already performed using the split-luciferase system [4]. This method of course, requires a cloned or clonable library ORF set. While this worked for the split-luciferase as the detection of luciferase activity has good signal-to-noise ratios, the BiFC system relies on weaker mYFP emission, and this emission must exceed that of the plant autofluorescence. Thus, we found that the screening was only possible using a flow cytometer where we can compare YFP versus autofluorescence to detect those cells expressing BiFC of YFP.

Due to the chanciness of the protoplast transfection and the DNA recovery, we choose to stay with the recovery of the plasmid DNA first. At the beginning of the screen design, we had anticipated that we would be able to isolate a single bacterial colony carrying a single plasmid type that could be screened pair-wise against the bait and that plasmid’s cDNA would be available for immediate downstream cloning via Gateway^TM^ technology. Unfortunately, although only few bacterial colonies were obtained, those that were obtained also carried multiple, independent plasmids as well. Furthermore, the majority of colony forming units derived by plasmid recovery from the protoplasts did not have interacting prey (Figure4B). This suggested to us that although the plasmid recovery from positive BiFC cells sorted by FACS did lead to a slight enrichment of the BiFC generating plasmids (Figure4E), other plasmids were still present in those protoplasts and also consequently in the bacteria. Our solution to the multiple-plasmid problem was an enrichment of the actual plasmids encoding the interacting protein by isolating all of the plasmids from the first round and transfect them against the bait once again and sort again by FACS on BiFC of YFP. This strategy worked, as the positive interaction rate went up from 2% to 27%.

In the end we utilized the protoplast transfection and the recovery of the bacterial plasmid DNA without any additional interventions to identify novel bait interacting partners and recovered the cDNA by PCR and cloning. Together these observations mean that the screen is not saturated. For example, the one positive found in the first round of the CPK3 screen was not found in the second round (Figure4). This also indicated that there were probably many more plasmids still present encoding for other CPK3-interacting partners that had been not detected. Nevertheless, we conclude that it is possible to recover interacting proteins encoded on bacterial plasmids from an *in planta* screen.

The majority of observed BiFC signals was surprisingly weak and only detectable in the flow cytometer. One would have expected that some strong BiFC interactions should have been detected as it was the case for APX3. The best explanation for the low BiFC-YFP signals during the screening is that, in order for enough YFP signal to be detected, it must exceed a certain concentration in the cell before the total fluorescence intensity is detectable above the autofluorescence. Reducing the YFP by using BiFC fragments already reduces the total detectable protein [15]. For those fusion proteins whom are less abundant in a cell or have localizations that restrict their abundance (for example those in the nucleus, plasma membrane or Golgi apparatus), it means that it is not possible to screen for any type of protein. It is not only remarkable that could we recover transfected plasmids from plant protoplasts after 36 hrs, but that it was also possible to identity some interacting partners with a bait construct. According to the data presented here, the *in vivo in planta* BiFC screen provides a lucrative alternative to search for novel protein-protein interactions that can, according to our data, only be found *in planta.*

### Putative interaction partners of CPK3

We choose CPK3 (AT4G23650) for demonstrating the screening method. CPK3 (and CPK6) are expressed not only in stomata but also in other tissues and are well studied for their function in guard cells and ABA signaling [36]. CPK3 is involved in the phosphorylation of plasma membrane S-type anion channels for the Ca^2+^-dependent stomatal closure [36], senses Ca^2+^ directly [37] in addition to regulating ROS signaling [36], which is also needed for ABA signaling [38]. CPK3 is further implicated in mediated ABA stomatal regulation involving phosphoinositides and differential Ca^2+^ mediations [39]. Putative phosphorylation targets of CPK3 were recently published [23] and the results show that potential CPK3 target proteins can be seemingly varied in functional classes. Hence, CPK3 is has been shown to be involved in many processes in addition to guard cell signaling [23,40]. CPK3 has been broadly localized to the cytoplasm and in and around the nucleus [22], but has recently been shown to be preferentially vacuolar and plasma membrane-associated [23]. Our CPK3 constructs were localized to the cytoplasm and in and around the nucleus in tobacco leaves and in *Arabidopsis* protoplasts (mYFP, Figure1; eGFP, Figure6) very much like that observed by [23]. Unfortunately, the BiFC CPK3-prey interactions were too weak to be observed in the microscope; thus we were not able to show where the BiFC interactions were taking place inside the cell. We used two fluorescence based methods to substantiate the protein-protein interactions found in the screen: biased BiFC and FRET-FLIM. I wanted to say that theoretically BIFC interactions could be through an unseen partner or due to trapping, but this sentence ended up a bit self-contradictory the way is it currently written. We used GFP/mCherry or GFP/mRFP donor/acceptor FRET pairs that have been shown to perform very well *in vivo*[35,41]. Proteins (and their fluorophore fusion) must be within 1 to 10 nm distance for FRET to occur [42], which is the typical distance found for interacting proteins. Similarly, BiFC has been discussed to occur over a distance around 7 nm [43][44]. Both BiFC and FRET-FLIM support four previously uncharacterized protein interactions of CPK3.

APX3 (#1, AT4G35000) showed the strongest BiFC with the CPK3 baits in both orientations, N-terminal and C-terminal SPYNE (Figure5C). APX3 is targeted to peroxisomes [24,45], but has been shown to be retarded in the cytoplasm by AKR2A [46]. In the FRET-FLIM studies, the N-terminal mRFP-APX3 fusion showed the strongest FRET efficiency with CPK3-eGFP (Figure6D). mRFP-APX3 was clearly non-homogenous in its sub-cellular distribution as its C-terminal transmembrane domain [24] was not masked. In contrast, the C-terminal APX3-mCherry was mis-localized to the cytoplasm (Figure6B; Additional file 3) and showed no interaction with CPK3 in FRET-FLIM (Figure6E). This evidence combined with the very strong BiFC makes a good argument that the screen was able to find a major interactor of CPK3. Interestingly, APXs are important for scavenging ROS (H_2_O_2_) and APX3 could provide the link proposed for CPK3 and CPK6 in regulating ROS and NADPH activation in guard cell function [36].

ORP2A (#5, AT4G22540) is a predicted oxysterol binding protein (OSBP). ORP2A significantly interacted with CPK3 in BiFC experiments (Figure5C). It also interacted preferentially with CPK3 in tobacco epidermal cells as shown by FRET-FLIM. OSBPs are involved in sterol trafficking [47] affecting membrane fluidity and permeability and influencing secretory events. OSBPs are known to bind to oxysterols, which compose minor amounts of sterols in plants [48], but OSBPs are known in other species to bind to different lipids including phosphoinositides, ergosterol, and cholesterol (references in [28]). Interestingly, there is some evidence that OSBPs are involved in the regulation of processes like Ca^2+^ uptake and transcriptional control, both processes which relate directly to CPK3. Mechanisms how newly synthesized sterols reach the plasma membranefrom the ER are unclear in plants and OSBPs are possible candidates. ORP2A is well expressed in many tissues and is somewhat regulated by stresses [28]. CPK3 was shown to target ER associated proteins Calnexin and Calreticulin [23], the latter of which regulates Ca^2+^ stores and signaling from the ER.

AT2G39050 (#6) is a ricin B-related lectin domain containing protein. Ricin is a heterodimeric plant protein that is toxic to mammalian and many other eukaryotic cells by binding to membrane localized galactose-containing receptors [29]. Ricin is composed to two subunits, ricin toxin A (RTA) and B (RTB). RTA is catalytically-active and removes a specific residue from the 28 S ribosomal RNA [49]. During its synthesis in plant cells ricin traffics from the ER, via the Golgi complex, to the vacuole [29]. AT2G39050 showed significant interaction with CPK3 in BiFC. The FRET-FLIM studies also support the interaction of this protein with CPK3. That CPK3 is also membrane associated and that ricin moves through the ER to the vacuole strongly supports the interaction with CPK3.

HMGB5 (AT4G35570) belongs to the class of high mobility group (HMG) proteins and are, after histones, the second most abundant type of chromosomal proteins [50]. HMGs have an ‘AT-hook’ that binds to the minor groove of short stretches of A/T-rich B-form DNA independent of the nucleotide sequence [51]. Unlike histones, HMG proteins are very dynamic and some even shuttle in and out of the nucleus in animal and plant cells [50,52]. HMGB5 is predominantly found inside the nucleus [30] and is extremely mobile within the nucleus [52]. HMGB5 showed a significant BiFC interaction with CPK3 in *Arabidopsis* protoplasts. The FRET-FLIM experiments in tobacco epidermal cells also substantiate the interaction between CPK3 with HMGB5 in the nucleus.

Among CPK3’s roles in the regulation of plasma membrane-localized ion-channels, it is known to have roles in phosphorylating nuclear transcription factors [32,53,54], other DNA-binding proteins [23,55]) and many RNA associated proteins [23]. This suggests that CPK3 has a role in regulating gene expression before, during and after transcription and that it may also have a role in chromatin regulation in conjugation with, for instance, HMGB5.

We could exclude AT2G29670 (#2), AT5G08680 (#3), GLO1 (#4, AT3G14420) and CCoAMT (#8, AT1G67980) as true interaction partners for CPK3 as they did not show interaction any via BiFC in *Arabidopsis* protoplasts where the screen was conducted and therefore we conclude they do not meet in *Arabidopsis* cells and thus do not interact with each other (see Additional file 5 and 6 for details).

## Conclusions

A new role for CPK3 and ROS regulation can now be hypothesized through the *bona fide* interaction with APX3. The combination of BiFC and FRET-FLIM measurements also validates the interaction of CPK3 with HMGB5, ORP2A, and AT2G39050 (Ricin-B). None of these interactions have been observed before. Our approach, therefore, is one of the first *in planta* random library screens shown to work. Although this method is not suited for high-throughput screens, it still is an alternative to search for novel interactions that may or may not be caught with other screening methods, as in our case with the yeast-two-hybrid system. And, as the screen is *in planta*, one still has the opportunity to treat the cells with elicitors, hormones or pharmaceuticals, as well as use protoplasts from mutant plant lines to screen for interactions that maybe dependent on such conditions.

## Methods

### Protoplast transfections

Protoplasts were transformed either in a large-scale (7.5 x 10^5^ to 1 x 10^6^ protoplasts per transfection) or in small-scale (6.0 x 10^4^ protoplasts per transfection). Protoplasts were generated from 3-day-old *Arabidopsis* Col-0 cell suspension culture. The suspension culture was maintained in a 250–300 ml Erlenmeyer flask, in the dark, at 24°C-26°C and 120 rpm (constant shaking) in 50 ml MSCol Medium (0.43% w/v MS salts, 0.1% w/v Nicotin acid, 0.1% w/v Pyridoxin-HCl, 1% w/v Thiamin-HCl, 10% w/v myo-Inositol, 3% w/v sucrose, pH = 5.8 with KOH and 0.1% v/v 2,4-D added after autoclaving). The protoplasts were prepared and transformed by the PEG method according to the protocols of [56,57], which were recently summarized and described by us in full detail in [58].

Large-scale transformations were performed as described in [12,58] in 14 ml round-bottom Falcon tubes. In short, the cells were collected by centrifugation (max. 100 g) and the cell walls are removed by incubation in cell wall digestion solution (1% cellulase, 0.25% macerozym, 8 mM CaCl_2_ , 0.4 M mannitol, pH 5.5, filter sterilized) for 6 hrs. The cells are washed and resuspended in W5 (154 mM NaCl, 125 mM CaCl_2_, 5 mM KCl, 5 mM glucose, pH 5.8–6.0, autoclaved) and kept at 4°C for 20–30 min. Thereafter the cells are transferred to MMM (15 mM MgCl_2_, 0.1% MES, 0.5 M mannitol, pH 5.8, autoclaved) and ready for PEG transfection. The cells were transfected with 20 ug DNA in water and PEG solution (40% PEG 4000, 0.4 M mannitol, 0.1 M Ca(NO_3_)_2_ , pH 8–9 (the pH needs 1–2 h to stabilize), autoclaved). After the transfection process, the transfected cells were stored in the dark at 24°C-26°C in standard K3 protoplast medium (see [58] for details of K3 medium preparation) before analysis (16 to 36 hrs later).

Small-scale transformations were performed in 96 well round-bottom PP plates (Roth) using the protocol described here. Protoplasts were generated as in [58], but once in W5 medium, were incubated for 30 min at 4°C and either processed immediately or stored overnight at 4°C. The cells were sieved through a 70 μM filter (Becton-Dickinson), re-counted and resuspended to 2 x 10^6^/cells/ml in MMG media (0.4 M mannitol, 15 mM MgCl_2_). CsCl purified plasmid DNA or the equivalent was added to each well (up to 5 μg) in a volume of 9 μl, followed by 30 μl of filtered protoplast cells (6 x 10^4^), and mixed well by gently knocking the palm against the plate about 10 times on each side. Once mixed, 30 μl of PEG solution (6.75 mM Ca(NO_3_)_2·_4H_2_O, 270 mM mannitol, 17.5 ml H_2_O, 38.5 % w/v PEG1500, [pH9.5 with 0,1 mM KOH], filter sterilized and stored in small aliquots) was added to each well and mixed thoroughly as just described and incubated for 10 min on the bench. Thereafter, 30 μl of MMG is added to each well and mixed completely as described, followed by quick addition of 250 μl of standard K3 protoplast medium [58] and mixed thoroughly for approx. 30 s or until any precipitated DNA had been dissolved. The plate was then covered with Nescofilm (Fisher Scientific) and incubated overnight at 26°C before analyses. For large-scale or high-throughput analyses, plasmid DNA was prepared with commercial DNA purification columns; for screening of putative interactors, standard mini-DNA preparations [59] or mini-DNA preparation kits were used. Protoplasts were incubated for 16 to 36 hrs before flow cytometric analysis or FACS; longer incubation times resulted in more positive signals and were necessary for detecting BiFC library interactions. BiFC fluorescence index was calculated as in [12], and statistical tests were performed in JMP9 (SAS).

### Transient expression in tobacco leaves

A single colony of transformed *Agrobacterium tumefaciens* was inoculated in 5 ml of YEB-Medium (0.5% beef extract, 0.5% sucrose, 0.1% yeast extract, 0.05% MgSO_4_·7H_2_O) containing Rif/Gent/ and vector-specific antibiotic at 28°C overnight. In the morning, 1 ml of the pre-culture was taken and re-inoculated into 5 ml of the same Medium. The same was done for *Agrobacterium* strain carrying the p19 RNAi-suppressor protein from tomato bushy stunt virus [60]. Each culture was collected in a 15-ml Falcon Tube and centrifuged at 4000 rpm for 20 min. Bacteria pellets were then resuspended in AS-Medium (10 mM MgCl_2_, 10 mM MES [5.6], 150 μM acetosyringone) to an optical density at 600 nm of about 0.7-0.8. The resuspended bacteria (two potential interaction partners and p19 strain) were mixed 600 ml each, a 1:1:1 ratio, in a 2 ml Eppendorf tube and incubated for 0.5-1 hour at 4°C.

*Nicotiana benthamiana* plants were cultivated in the greenhouse on soil with 60% humidity, a 14 h light peroid, and a 25°C day/19°C night temperature cycle. The bacterial solution was inoculated into the entire leaf area through the abaxial sides using a 1-ml syringe; two leaves per plant were inoculated. After inoculation, the plants were kept in a tray with a hood at 25°C. Two days after the bacterial inoculation, the pABind vectors [34] and the N-terminal mFRP vector pB7WGR2,0 (Plant Systems Biology, Gent) were induced by application of estradiol by brushing a 20 μM estradiol (in 0.1% Tween-20) solution onto the abaxial leaf surface. FRET measurements were performed 24 to 48 hours after estradiol application.

### FACS

FACS and flow cytometric analyses were performed with a MoFlo (2007; Beckman-Coulter). mGFP, mYFP, mRFP or mCherry were excited with a 50 mW 488 nm argon laser. GFP and YFP were detected in FL1 (510 – 550 nm), Auto-fluorescence in FL2 (565 – 605 nm) and RFP/mCherry in FL3 (605 – 650 nm). RFP and mCherry expression was cross checked with a 50 mW 532 nm solid-state laser and detected behind a 585/30 bandpass for RFP or 613/20 for mCherry. Transfected protoplasts were sieved through 40 μM (BD) before FACS or 70 μM filters before analysis. Sorts and analyses were run approximately under 31.0/30.0 psi (sample/sheath) using a 100 μM nozzle; sheath was 1x PBS at pH 7.0.

Protoplasts identified with a detectable BiFC signal were sorted directly into Edwards’s Buffer [14] in a 2 ml eppendorf tube at 20°C. The collected protoplasts were thoroughly mixed, then iso-propanol was added 1:1, incubated at −20°C for 30’, centrifuged at 13000 rpm at 4°C for 45’, washed with cold 75% ethanol, centrifuged for at 4°C for 15’. The resultant precipitate was air-dried for 15’ on the bench at room-temperature and resuspended in 20 μl Millipore purified water. 50 μl chemically competent NEB 10-beta (New England Biolabs) cells were thawed on ice and added to 10 μl DNA extraction, further incubated for 30’ followed by a 30” heat-shock at 42°C, then 2 min on ice, and incubated for 2 hrs at 37°C in 800 μl SOC medium. The transformation was then plated out on two large (145 x 20 mm) petri dishes containing LB-agar with 100 μg /ml ampicillin to select for colonies with the pE-SPYCE-cDNA library vector. To recover plasmids from colonies on a plate, the bacteria were mixed and removed in 10 ml LB with selection antibiotic added to the plate. The plasmid DNA was then purified using commercial midi-DNA preparation columns.

### Cloning

35S::cDNA::YN173AcV5 (Spec^R^) constructs were constructed with multi-site GW vectors (pEntryL4R1-P35S, pH7m34GW or pB7m34GW) from Plant Systems Biology (Gent). The pENTR-R2L3-YN173AcV5 was generated by amplifying from N-terminus until the 173 amino acids (fwd: B2 + gaATGGTGAGCAAGGGCGAG and rev top strand (YFP, AcV5, stop): CGCCACAACATCGAGGAC**-**TCTTGGAAAGATGCGAGCGGCTGGTCTTGAt + B3) of mYFP by PCR and cloned via BP-reaction into pDONRP2R-P3 (Invitrogen). pUC-SPYC/NE-mRFP vecrtors are described in [12]; pUC SPYC/NE vectors are those described in [6]; pPE-SPYC/NE::cDNA library was made by cloning the pSPORT-P (Kan^R^) *Arabidopsis* cDNA library (Supescript *Arabidopsis* third-flower stage seedlings #11474012) into pDONR222 (resultant titer 5.1 x 10^6^ cfu/ml) and recombining it with pE-SPYCE/SPYNE ([61]; Amp^R^) vectors to make YFP-fragment fusions (resultant titers: pE-SPYNE-cDNA at 3.36 x 10^6^ cfu/ml; pE-SPYCE-cDNA at 3.95 x 10^6^ cfu/ml). The libraries were grown on 30 cm diameter LB plates plus ampicillin at 28°C for 18 hrs. The bacteria were harvested from the plates using LB liquid plus antibiotic followed by plasmid purification using a Qiagen Giga-prep kit following the manufacturer’s instructions.

After sequencing interacting partner clones in pE-SPYCE, the matching cDNAs were cloned from cDNA produced from Arabidopsis (Col-0) leaf material. The full-length cDNAs were cloned into either pENTR/D-TOPO (Invitrogen) or pDONR207 (Invitrogen) and subsequently recombined into target Destination vectors as needed. FRET-FLIM vectors were pABindGFP / mCherry / GFP::mCherry [34] as C-terminal tag fusions. N-terminal mRFP fusions were made with pB7WGR2.0 (Plant Systems Biology, Gent). Additional clones not mentioned explicitly were provided by collaborators and are listed in the Acknowledgements.

### FRET-FLIM and microscopy

FRET-FLIM measurements were carried out as previously described in [35] with the addition that the presence of the acceptor was confirmed. Statistical tests were performed in JMP9. Confocal Laser Scanning Microscopy was performed as described in [35].

### Yeast-two-hybrid

Yeast two-hybrid experiments were performed using the Matchmaker™System (Clontech). Plasmids were constructed by LR-reaction of corresponding Entry clones and destination vectors pGBKT7-DEST or pGADT7-DEST [62]. Yeast strain PJ69-4A was transformed using lithium acetate/SS-DNA/PEG method [63]. After 3 days of growth on vector selective media (CSM, -L, -W), 6 independent clones were picked, resuspended in ddH2O and 10 μl were dropped on vector-selective media. Subsequently, 10 μl of culture were dropped on vector- and interaction-selective media (CSM, -L, -W-, -A) and incubated at 28°C. At day 3 the growth of the clones was monitored. In addition, yeasts from selective media were inoculated in selective media (CSM,-L,-W) harvested and analyzed by western-blot to determine the correct expression of the fusion proteins [63].

## Competing interests

The authors declare that they have no competing interests.

## Authors' contributions

KWB and KH designed, conducted and were funded to perform the experiment. KWB, MB, NW, YZ, FKET, and MV performed the molecular laboratory work. FS and SP built the custom-built confocal stage scanning microscope and conducted the *in vivo* FRET-FLIM measurements. All authors read and approved the final manuscript.

## Supplementary Material

Additional file 1 **Phred DNA trace files of original prey plasmids.** DNA trace files from the original plasmid mini-preps that encoded for proteins that theoretically interacted with the bait protein. Click here for file

Additional file 2 **Significance Tests for BiFC and FRET-FLIM quantifications.** P-values for all significance tests that were mentioned in the text or in figure5 and 6. Click here for file

Additional file 3 **Confocal Localization Images of prey fusion proteins with mCherry or mRFP.** Confocal images of prey fusion proteins expressed in tobacco epidermal cells with enlarged insets of APX3 fusions. Click here for file

Additional file 4 **Yeast-two-hybrid assays of CPK3 with prey proteins.** Representative yeast-two-hybrid assays performed with CPK3 verses prey proteins on selective and non-selective media. Six independent colonies were analyzed per combination. Western blots are also shown for all proteins and the band corresponding to the full-length protein is indicated with an asterisk. Click here for file

Additional file 5 **Additional discussion text for non-interacting prey proteins. **Additional discussion is provided for the four prey proteins that we consider not to be interaction partners of CPK3. Click here for file

Additional file 6 **Confocal Localization Images of CPK3-eGFP with tetra-trico peptide repeat (TPR) AT2G29670.** Confocal images of of CPK3-eGFP with tetra-trico peptide repeat (TPR) illustrating conglomerates of AT2G29670 that led to the exclusion of CPK3-eGFP. Click here for file
