# Supplementary material for: Screening for in planta protein-protein interactions combining bimolecular fluorescence complementation with flow cytometry
Source: Plant Methods. 2012 Jul 12;8:25. doi: 10.1186/1746-4811-8-25 (PMC3458939; doi:10.1186/1746-4811-8-25)
Supplement: Additional file 2 — Significance Tests for BiFC and FRET-FLIM quantifications. P-values for all significance tests that were mentioned in the text or in figure5 and 6. [file 1746-4811-8-25-S2.pdf]

Add. File 2. Significance Tests
Significance tests were conducted with JMP9(SAS) after import using an alpha level of 0.5.
Those that are significantly different from the control are highlighted in bold.

CPK3-eGFP vs mRFP-XP alpha =0.5

cytoplasm Student's t

Level				P-value (with respect to CPK3)
CPK3-GFP	A			1
CCoAMT	A	B		0.0632
GLO1		B		**0.0416**
AT2G39050		B		**0.0384**
AT5G08680		B		**0.0067**
ORP2A		B		**0.0031**
AT2G29670		B		**0.002**
APX3			C	**0.0001**

cytoplasm Dunnett's Method

Level	LSD	P-value (with respect to CPK3)
CPK3-GFP	-0.08	1
CCoAMT	-0.02	0.2709
GLO1	-0.02	0.1896
AT2G39050	-0.02	0.1769
AT5G08680	0.004	**0.0362**
ORP2A	0.012	**0.0178**
AT2G29670	0.016	**0.0116**
APX3	0.095	**0.0001**

cytoplasm Tukey-Kramer HSD

Level				P-value (with respect to CPK3)
CPK3-GFP	A			1
CCoAMT	A	B		0.5451
GLO1	A	B		0.4226
AT2G39050	A	B		0.4012
AT5G08680	A	B		0.1058
ORP2A	A	B	C	0.0553
AT2G29670		B	C	**0.0372**
APX3			C	**0.0001**

nucleus Student's t

Level			P-value (with respect to CPK3)
CPK3-GFP	A		0.5
HMGB5		B	**0.0084**

nucleus Dunnett's Method

Level	LSD	P-value (with respect to CPK3)
CPK3-GFP	-0.14	1
HMGB5	0.088	**0.0056**

nucleus	Tukey-Kramer HSD			
	Level			P-value (with respect to CPK3)
	CPK3-GFP	A		
	HMGB5		B	**0.0084**

CPK3-eGFP vs XP-mCherry alpha =0.5

cytoplasm Student's t

Level						P-value (with respect to CPK3)
CPK3-GFP	A					1
APX3	A	B				0.0761
HMGB5	A	B	C			0.0665
CCoAMT			C			**0.0069**
AT5G08680			C	D		**0.0001**
AT2G29670		B	C	D		**0.005**
ORP2A			C	D		**0.0001**
GLO1			C	D		**0.0001**
AT2G39050				D		**0.0001**
CPK3-FRET					E	**0.0001**

cytoplasm Dunnett's Method

Level	LSD	P-value (with respect to CPK3)
CPK3-GFP	-0.04	1
APX3	-0.02	0.4297
HMGB5	-0.02	0.3874
CCoAMT	-4.00E-04	0.0535
AT5G08680	0.019	**0.001**
AT2G29670	0.002	**0.0392**
ORP2A	0.037	**0.0001**
GLO1	0.031	**0.0001**
AT2G39050	0.047	**0.0001**
CPK3-FRET	0.444	**0.0001**

cytoplasm Tukey-Kramer HSD

Level					P-value (with respect to CPK3)
CPK3-GFP	A				1
APX3	A	B			0.7401
HMGB5	A	B	C		0.7001
CCoAMT	A	B	C		0.1669
AT5G08680		B	C		**0.0043**
AT2G29670	A	B	C		0.1282
ORP2A		B	C		**0.0001**
GLO1		B	C		**0.0006**
AT2G39050			C		**0.0001**
CPK3-FRET				D	**0.0001**

nucleus	Student's t			
	Level			P-value (with respect to CPK3)
	CPK3-GFP	A		1
	HMGB5		B	**0.0001**

nucleus	Dunnett's Method		
	Level	LSD	P-value (with respect to CPK3)
	CPK3-GFP	-0.04	1
	HMGB5	0.068	**0.0003**

nucleus	Tukey-Kramer HSD			
	Level			P-value (with respect to CPK3)
	CPK3-GFP	A		1
	HMGB5		B	**0.0001**

BIFC CPK3-YN173 alpha =0.5

cytoplasm	Student's t				
	Sample	Level			P-value (with respect to CPK3 alone)
	APX3	A			**0.0001**
	ORP2A		B		**0.0007**
	AT2G39050		B		**0.0030**
	HMGB5		B		**0.0051**
	CCoAMT			C	0.8414
	AT5G08680			C	0.8414
	AT2G29670			C	0.8870
	CPK3alone			C	1.0000
	GLO1			C	0.9924

cytoplasm	Dunnett's Method		
	Sample	Abs(Dif)-LSD	P-value (with respect to CPK3)
	APX3	443.3	**0.0001**
	ORP2A	15.64	**0.0044**
	AT2G39050	6.672	**0.0181**
	HMGB5	3.396	**0.0300**
	CCoAMT	-34.1	0.9993
	AT5G08680	-36.8	1.0000
	AT2G29670	-37.6	1.0000
	CPK3alone	-39.5	1.0000
	GLO1	-39.4	1.0000

BIFC YN154-CPK3 alpha =0.5

cytoplasm	Student's t			
	Level			P-value (with respect to CPK3)
	APX3	A		**0.0001**
	HMGB5		B	0.3602
	AT2G39050		B	0.5240
	ORP2A		B	0.6336
	AT5G08680		B	0.9584
	CCoAMT		B	0.9730
	CPK3alone		B	1.0000
	GLO1		B	0.9969

cytoplasm	Dunnett's Method		
	Level	Abs(Dif)-LSD	P-value (with respect to CPK3)
	APX3	324.5	**0.0001**
	HMGB5	-28.7	0.8811
	AT2G39050	-32.9	0.9774
	ORP2A	-35.3	0.9955
	AT5G08680	-41.6	1.0000
	CCoAMT	-41.9	1.0000
	CPK3alone	-42.4	1.0000
	GLO1	-42.3	1.0000
