# Supplementary figures and images for: Screening for in planta protein-protein interactions combining bimolecular fluorescence complementation with flow cytometry
Source: Plant Methods. 2012 Jul 12;8:25. doi: 10.1186/1746-4811-8-25 (PMC3458939; doi:10.1186/1746-4811-8-25)

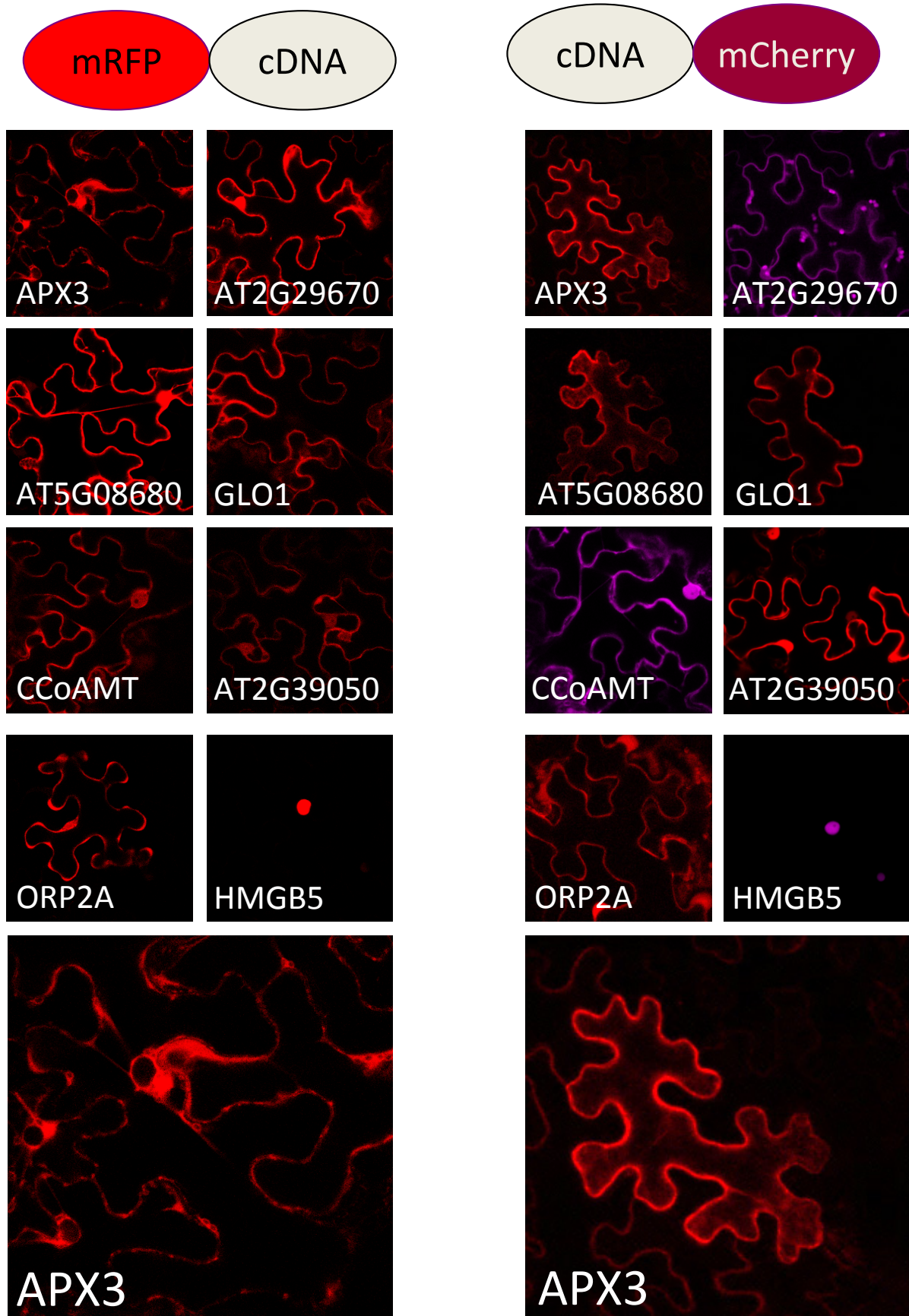

Add. File 3. Confocal images of fish fused to N-terminal mRFP or C-terminal mCherry.

Supplement: Additional file 3 — Confocal Localization Images of prey fusion proteins with mCherry or mRFP. Confocal images of prey fusion proteins expressed in tobacco epidermal cells with enlarged insets of APX3 fusions. [file 1746-4811-8-25-S3.pdf]
