# Supplementary material for: Screening for in planta protein-protein interactions combining bimolecular fluorescence complementation with flow cytometry
Source: Plant Methods. 2012 Jul 12;8:25. doi: 10.1186/1746-4811-8-25 (PMC3458939; doi:10.1186/1746-4811-8-25)
Supplement: Additional file 4 — Yeast-two-hybrid assays of CPK3 with prey proteins. Representative yeast-two-hybrid assays performed with CPK3 verses prey proteins on selective and non-selective media. Six independent colonies were analyzed per combination. Western blots are also shown for all proteins and the band corresponding to the full-length protein is indicated with an asterisk. [file 1746-4811-8-25-S4.pdf]

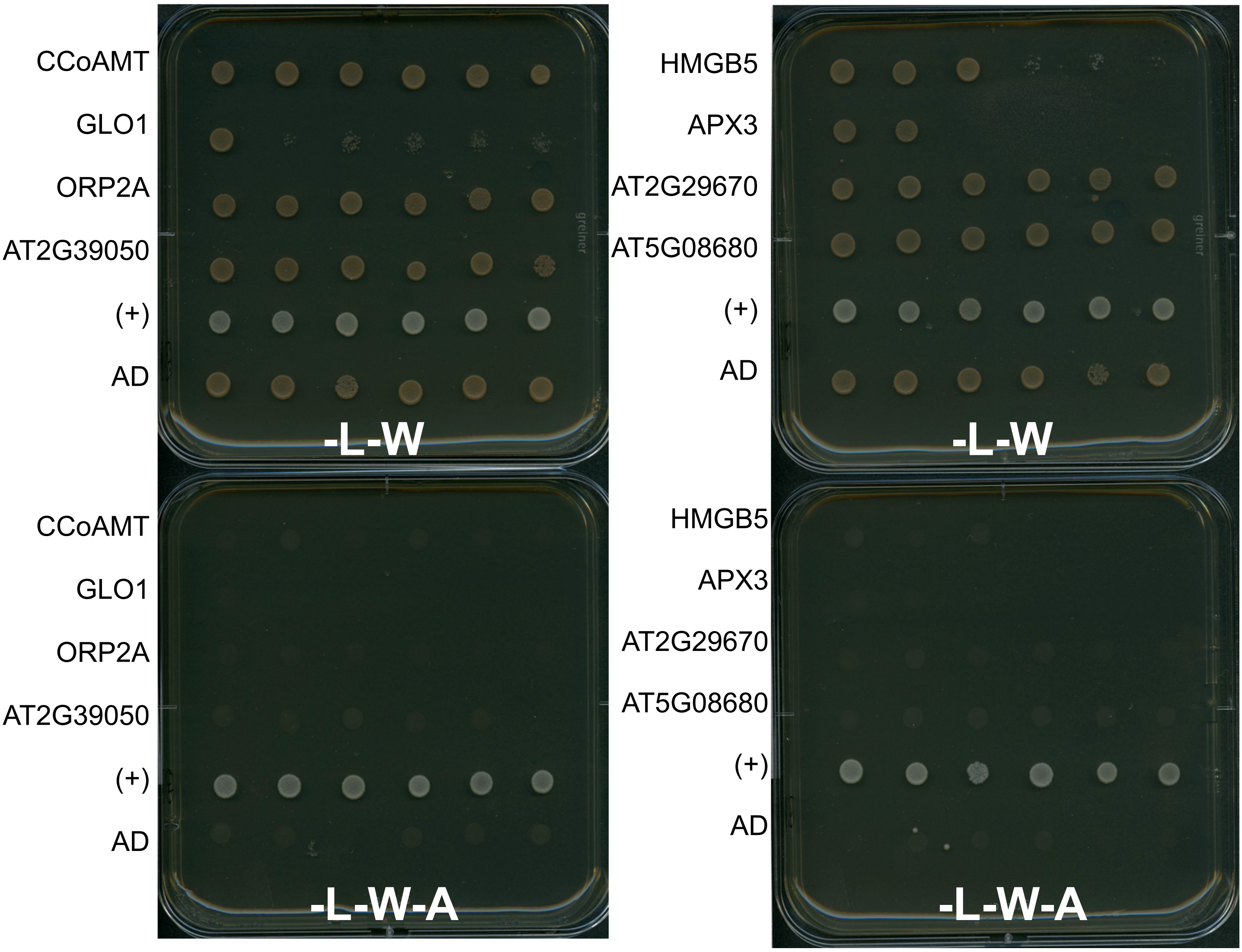

LEGEND

1. BD-CPK3
2. HMGB5
3. APX3
4. At2g29670
5. At5g08680
6. CCoAMT
7. GLO1
8. ORP2A
9. At2g39050
10. Empty-AD
(+). positive
(-). no plasmid

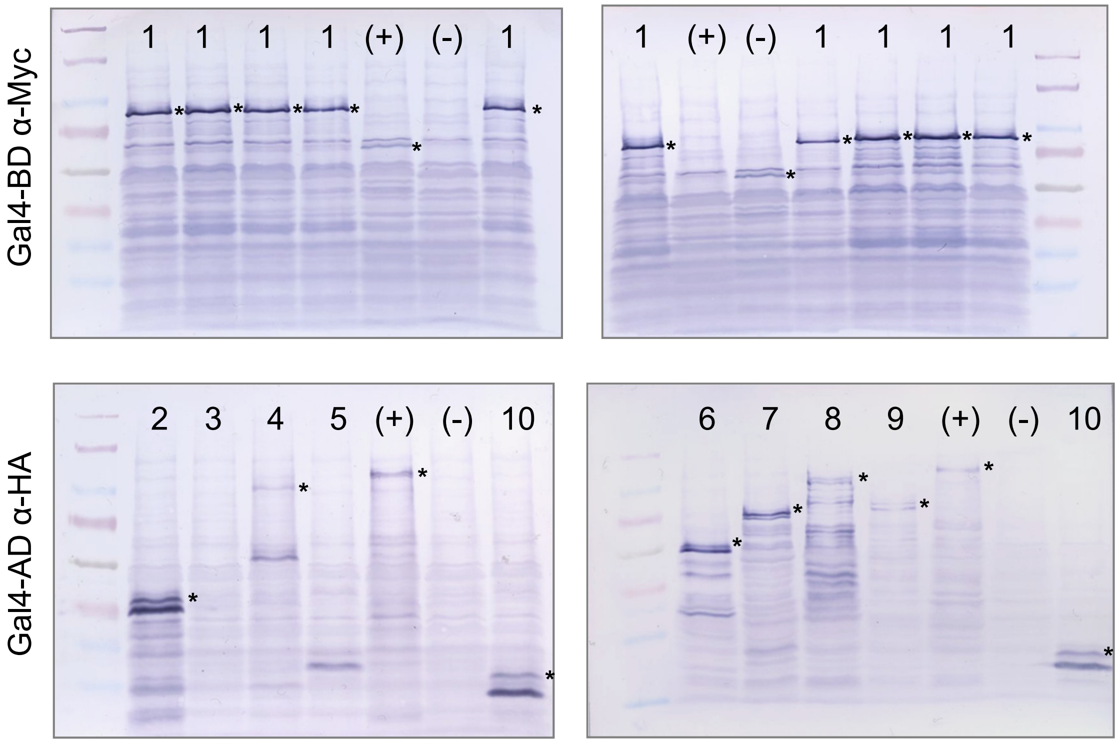

Add. File 4. Representative Yeast-two-Hybrid results. This experiment was repeated 4 times with the same result.
