# Supplementary material for: Screening for in planta protein-protein interactions combining bimolecular fluorescence complementation with flow cytometry
Source: Plant Methods. 2012 Jul 12;8:25. doi: 10.1186/1746-4811-8-25 (PMC3458939; doi:10.1186/1746-4811-8-25)
Supplement: Additional file 5 — Additional discussion text for non-interacting prey proteins. Additional discussion is provided for the four prey proteins that we consider not to be interaction partners of CPK3. [file 1746-4811-8-25-S5.pdf]

Additional file 5: Discussion of prey-proteins that did not interact with CPK3.

AT2G29670 (#2) encodes an uncharacterized protein that carries a tetra-trico peptide repeat (TRP) that mediates protein-protein interactions (D'Andrea and Regan 2003). TPR containing proteins are localized in a variety of subcellular compartment, including the nucleus, the cytoplasm and mitochondria and are involved in multiple processes (D'Andrea and Regan 2003). As such, TPR repeats mediate protein–protein interactions and the assembly of multi-protein complexes. Expression of AT2G29670-mCherry leads to the presence of aggregates that were composed purely of AT2G29670 (as judged by exclusion of even CPK3-eGFP (see Additional file 6). AT2G29670 interacted signficantly with CPK3 via FRET-FLIM measurements in both fusion orientations, but did not show any interaction with CPK3 via BiFC. Based on this observation it is more likely that they do not co-localize in Arabidopsis cells and, thus do not interact with each other.

AT5G08680 (#3) has been shown to be associated with mitochondrial (Heazlewood 2004) membranes as it is a β-subunit of mitochondrial ATP synthase. If it is interacting with CPK3 then it would be on the way to its destination however, from our localization data it is a seemingly free cytoplasmic protein. According to FRET-FLIM measurements At5g08680 associated weakly with CPK3 in both protein fusion orientations significantly based on the Students t-test and Dunnett's Method. The BiFC expression studies could not support this however. Based on these observations, it is more likely that they do not meet in Arabidopsis cells and thus do not interact with each other.

GLO1 (#4, At3g14420) is located in peroxisomes and is involved in the production of H_2O_2, the opposite of APX3. GLO1 glycolate 2-phosphate produced from photooxidation is oxidized to glyoxylate with the concomitant production of H_2O_2. Although there is some weak FRET-FLIM evidence that would suggest interaction with CPK3, our confocal images show that GLO1 is most likely mislocalized in tobacco epidermal leaf cells when it has a N-terminal mRFP or a C-terminal mCherry fusion. Furthermore, GLO1 absolutely did not show any BIFC signal with CPK3.

CCoAMT (#8, AT1G67980) is a completely uncharacterized S-adenosyl-L-methionine:trans-caffeoyl-coenzymeA 3-O-methyltransferase protein in Arabidopsis (Pakusch, Matern et al. 1991). It is predicted to play a role in the synthesis of feruloylated polysaccharides and in phenopropanoid biosynthesis and incorporate ferulic esters into cell walls. Three targets of alkaloid and phenylpropanoid biosynthesis were found targets of CPK3: a putative cinnamyl-alcohol dehydrogenase (CAD), an O-methyltransferase and strictosidine synthase 2 (SS2) (Mehlmer, Wurzinger et al. 2010). This makes CCoAMT a hot

candidate as an interacting partner of CPK3. Unfortunately, CCoAMT was not expressed and could not be determined to interact with CPK3 via BiFC in protoplasts. In addition, the FRET-FLIM results showed no significant reduction in the eGFP lifetime was observed for either CPK3 in either orientation, and thus is a false-positive.

References:

D'Andrea, L. D. and L. Regan (2003). "TPR proteins: the versatile helix." Trends Biochem Sci **28**(12): 655-662.

Heazlewood, J. L. (2004). "Experimental Analysis of the Arabidopsis Mitochondrial Proteome Highlights Signaling and Regulatory Components, Provides Assessment of Targeting Prediction Programs, and Indicates Plant-Specific Mitochondrial Proteins." The Plant Cell Online **16**(1): 241-256.

Mehlmer, N., B. Wurzinger, et al. (2010). "The Ca2+-dependent protein kinase CPK3 is required for MAPK-independent salt-stress acclimation in Arabidopsis." The Plant Journal **63**(3): 484-498.

Pakusch, A. E., U. Matern, et al. (1991). "Elicitor-Inducible Caffeoyl-Coenzyme A 3-O-Methyltransferase from Petroselinum crispum Cell Suspensions : Purification, Partial Sequence, and Antigenicity." Plant Physiol **95**(1): 137-143.
