# Supplementary material for: Screening for in planta protein-protein interactions combining bimolecular fluorescence complementation with flow cytometry
Source: Plant Methods. 2012 Jul 12;8:25. doi: 10.1186/1746-4811-8-25 (PMC3458939; doi:10.1186/1746-4811-8-25)
Supplement: Additional file 6 — Confocal Localization Images of CPK3-eGFP with tetra-trico peptide repeat (TPR) AT2G29670. Confocal images of of CPK3-eGFP with tetra-trico peptide repeat (TPR) illustrating conglomerates of AT2G29670 that led to the exclusion of CPK3-eGFP. [file 1746-4811-8-25-S6.pdf]

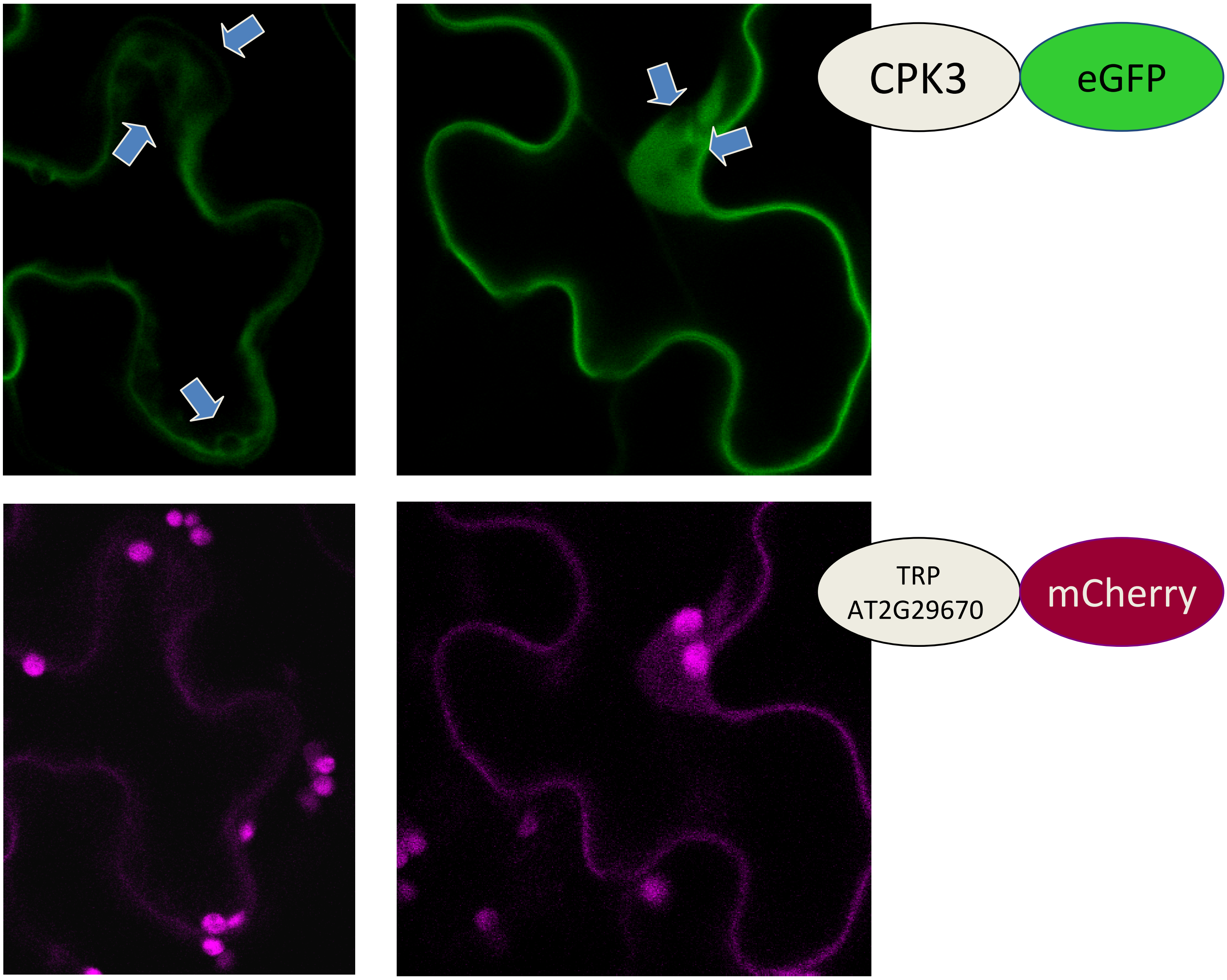

Add. File 6. Confocal image of the tetra-trico peptide repeat (TRP) containing protein TRP protein AT2G29670. Arrows show where conglomerates of AT2G29670 led to the exclusion of CPK3-eGFP.
