# Supplementary material for: Screening for in planta protein-protein interactions combining bimolecular fluorescence complementation with flow cytometry
Source: Plant Methods. 2012 Jul 12;8:25. doi: 10.1186/1746-4811-8-25 (PMC3458939; doi:10.1186/1746-4811-8-25)
Supplement: Additional file 1 — Phred DNA trace files of original prey plasmids. DNA trace files from the original plasmid mini-preps that encoded for proteins that theoretically interacted with the bait protein. [file 1746-4811-8-25-S1.pdf]

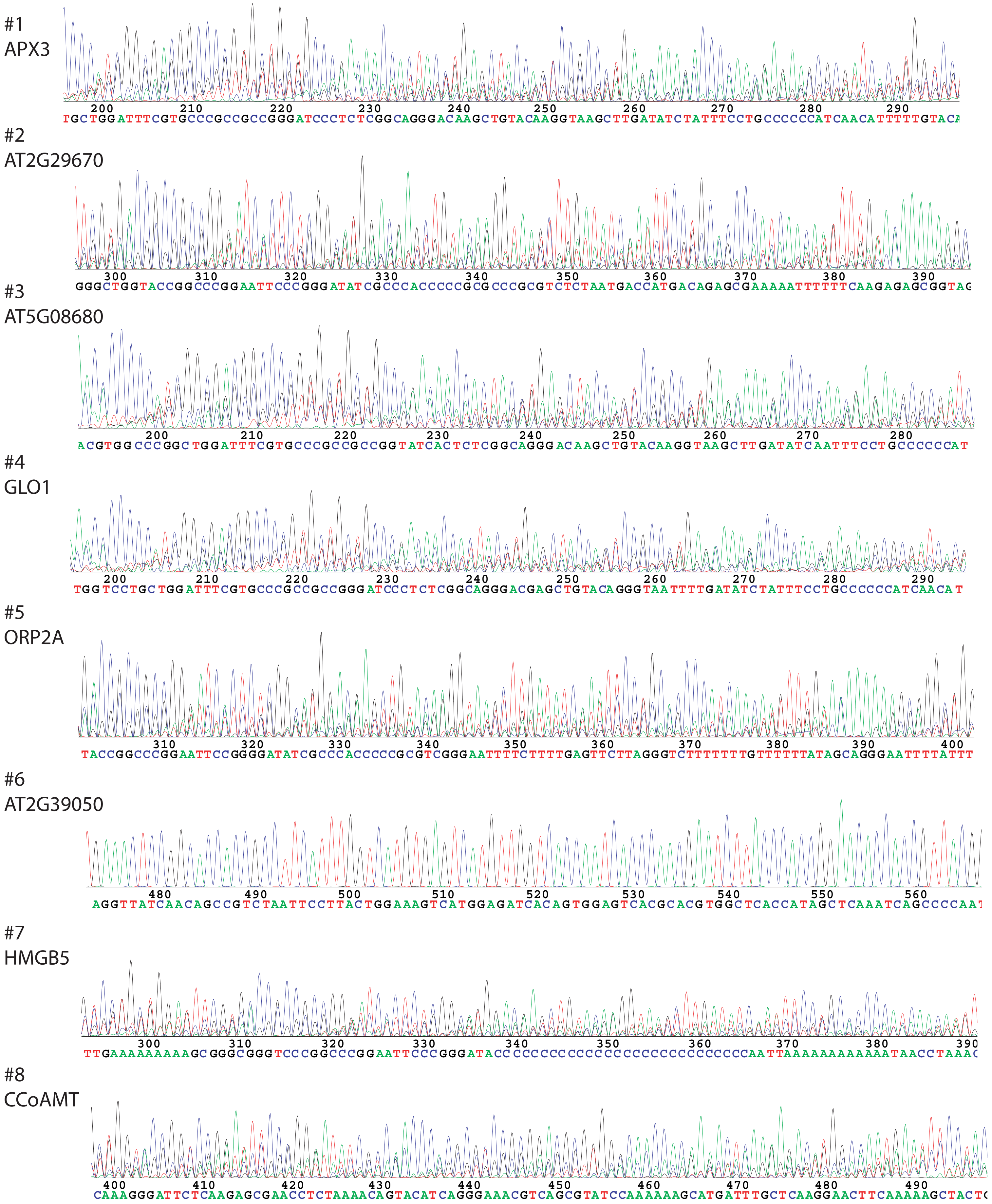

Add. File 1. Trace file excerpts from colonies that contained plasmids that interacted via YFP BiFC with CPK3-YN173.
